# Medicaid prescription limits: policy trends and comparative impact on utilization

**DOI:** 10.1186/s12913-016-1258-0

**Published:** 2016-01-15

**Authors:** Daniel A. Lieberman, Jennifer M. Polinski, Niteesh K. Choudhry, Jerry Avorn, Michael A. Fischer

**Affiliations:** 1 Division of Emergency Medicine, University of Washington, Seattle, WA USA; 2 CVS Health, Woonsocket, RI USA; 3 Division of Pharmacoepidemiology and Pharmacoeconomics, Brigham and Women’s Hospital and Harvard Medical School, Boston, MA USA

**Keywords:** Health policy, Medicaid, Prescription drugs, Pharmacy benefits, Pharmacoepimiology, Health services research

## Abstract

**Background:**

Medicaid programs face growing pressure to control spending. Despite evidence of clinical harms, states continue to impose policies limiting the number of reimbursable prescriptions (*caps*). We examined the recent use of prescription caps by Medicaid programs and the impact of policy implementation on prescription utilization.

**Methods:**

We identified Medicaid cap policies from 2001–2010. We classified caps as applying to all prescriptions (*overall caps*) or only branded prescriptions (*brand caps*). Using state-level, aggregate prescription data, we developed interrupted time-series analyses to evaluate the impact of implementing overall caps and brand caps in a subset of states with data available before and after cap initiation. For overall caps, we examined the use of essential medications, which were classified as preventive or as providing symptomatic benefit. For brand caps, we examined the use of all branded drugs as well as branded and generic medications among classes with available generic replacements.

**Results:**

The number of states with caps increased from 12 in 2001 to 20 in 2010. Overall cap implementation (*n* = 3) led to a 0.52 % (*p* < 0.001) annual decrease in the proportion of essential prescriptions but no change in cost. For preventive essential medications, overall caps led to a 1.12 % (*p* = 0.001) annual decrease in prescriptions (246,000 prescriptions annually) and a 1.20 % (*p* < 0.001) decrease in spending (−$12.2 million annually), but no decrease in symptomatic essential medication use. Brand cap implementation (*n* = 6) led to an immediate 2.29 % (*p* = 0.16) decrease in branded prescriptions and 1.26 % (*p* = 0.025) decrease in spending. For medication classes with generic replacements, the decrease in branded prescriptions (0.74 %, *p* = 0.003) approximately equaled the increase in generics (0.79 %, *p* = 0.009), with estimated savings of $17.4 million.

**Conclusions:**

An increasing number of states are using prescription caps, with mixed results. Overall caps decreased the use of preventive but not symptomatic essential medications, suggesting that patients assign higher priority to agents providing symptomatic benefit when faced with reimbursement limits. Among medications with generic replacements, brand caps shifted usage from branded drugs to generics, with considerable savings. Future research should analyze the patient-level impact of these policies to measure clinical outcomes associated with these changes.

**Electronic supplementary material:**

The online version of this article (doi:10.1186/s12913-016-1258-0) contains supplementary material, which is available to authorized users.

## Background

In 2010, Medicaid insured 51.5 million low-income Americans at a cost of almost $390 billion [1]. With the optional Medicaid expansion in the Patient Protection and Affordable Care Act (ACA) and budgetary shortfalls caused by the recent recession, states face increasing pressure to control costs for an expanding Medicaid population [2, 3]. Prescription drugs represent an inviting cost-control target for Medicaid, with $13.7 billion in spending in 2010 [1]. However, since many drugs are highly effective at decreasing morbidity and mortality, broad, untargeted limitations on drug availability may be a counterproductive strategy.

States have instituted multiple policies to control prescription costs, including limits on the number of reimbursable prescriptions (*caps*). Prior research has shown a substantial negative impact of caps. In 1981, New Hampshire implemented a three drug per month limit for Medicaid recipients, which lead to decreased use of essential medications, increased nursing home admissions, and increased use of emergency services by patients with schizophrenia [4–6]. Few recent studies have examined the impact of prescription caps [7, 8].

Despite previously demonstrated harms, Medicaid programs continue to implement prescription caps, including caps on branded prescriptions, a more novel approach [9]. Due to the Medicaid expansion, these cap policies already in place are affecting an increasing number of individuals. Given their expanding impact, understanding the effects of caps is critical for Medicaid pharmacy programs and more broadly for rational pharmaceutical benefit design.

We surveyed the use of prescription caps by Medicaid programs from 2001–2010 and used the natural experiment created by cap implementation to evaluate the impact of caps on prescription use and spending. We hypothesized that prescription use would shift away from preventive medications in favor of medications that provide symptomatic benefit in response to caps on total prescriptions. Additionally, we hypothesized that the use of branded drugs would decrease after implementation of caps on branded prescriptions and sought to determine whether there would be a corresponding increase in generic medication use.

## Methods

### Data collection

We gathered information on Medicaid policies placing limits on the number of prescriptions reimbursed (*caps*) in all 50 states and the District of Columbia from January 1, 2001 to December 31, 2010. Data sources included annual reference volumes compiled by the National Pharmaceutical Council, cross-sectional surveys from the Kaiser Family Foundation, state websites, and direct contact with state Medicaid offices [9, 10]. When sources conflicted, we assumed that information obtained directly from states was accurate. Additional file 1 (online Table S1) contains a list of resources organized by state. We extracted information on cap levels, drugs affected, recipients and medications excepted, and dates of policy changes. We classified caps as applying to all medications (*overall caps*) or branded medications only (*brand caps*).

We obtained prescription utilization data from the Center for Medicare and Medicaid Services (CMS), which provides quarterly data on aggregate drug use by state Medicaid programs [11]. These state-level data include the number of prescriptions filled, the number of medication units dispensed, and the Medicaid reimbursement for each medication, grouped by National Drug Code (NDC). We merged Medicaid data by NDC with the National Drug Data File from First Databank to include information on brand/generic status and therapeutic class [12]. We performed quality checks to identify erroneous entries (see Additional file 1, online methods). Non-publicly available information on manufacturer rebates to Medicaid programs is not included in these data. No patient-level data were included in analyses. The study was approved by the Brigham and Women’s Hospital institutional review board.

### Samples

We considered a subset of states that had data available for adequate time before and after cap initiation and implemented overall caps or brand caps as a sole intervention (see Additional file 1, online methods). In practical terms, this meant if a state already had a cap in place in 2001, we could not include it in analyses since we did not have pre-implementation data. This also meant that if a state simultaneously implemented both overall and brand cap policies, it could not be included in analyses because the effect of a single policy change could not be isolated. While this limited the sample size for the quantitative analysis, it did result in examination of a more homogeneous group of policies. States examined implementing overall caps (*n* = 3) included Louisiana (8 prescriptions monthly), Oregon (15 unique drugs in a 6 month period), and Utah (7 prescriptions monthly). States examined implementing brand caps (*n* = 6) included Alabama (4 branded prescriptions monthly), Illinois (3 branded prescriptions monthly), Kansas (5 branded prescriptions monthly), Kentucky (3 branded prescriptions monthly), Maine (5 branded prescriptions monthly), and Washington (4 branded prescriptions monthly). States implementing brand and overall caps were compared to states without caps. We excluded from the control group states that had cap policies at any point during the study period as well as states that had excessive missing or poor quality data, leaving the following 19 control states: Alaska, Connecticut, District of Columbia, Hawaii, Idaho, Indiana, Iowa, Maryland, Massachusetts, Minnesota, Michigan, Missouri, Nebraska, New Hampshire, New Jersey, North Dakota, South Dakota, Virginia, and Wisconsin.

### Outcomes

Based on published classifications, we defined a list of *essential medications* that prevent clinically significant morbidity or mortality for common conditions [4, 13, 14]. We excluded frequently overused medications, such as antiulcer medications, antidepressants, antibiotics, analgesics, non-steroidal anti-inflammatory drugs (NSAIDS), stimulants, anxiolytics, and some anti-epileptics often prescribed for neuropathic pain [15–18]. From these essential medications, we created a list of *symptomatic essential medications* that are likely to provide substantial symptomatic relief or prevent symptoms within a short timeframe after use; other medications were classified as *preventive essential medications*. Classifications were adapted from earlier publications [4, 13, 14]; final assignments were reviewed and agreed upon by consensus among physician coauthors (DL, NKC, JA, MAF).

*Symptomatic essential medications* included the following agents: antipsychotics, anti-parkinsonian agents, loop diuretics, short acting anti-anginal agents, short acting bronchodilators, long acting bronchodilators, oral corticosteroids, and anti-epileptics (excluding gabapentin, pregabalin, benzodiazepines, and barbiturates). *Preventive essential medications* included the following agents: anti-hyperlipidemics, anti-hypertensives, hypogylcemics, anti-coagulants, anti-retrovirals, anti-tubercular agents, anti-arrhythmics, bone resorption inhibitors, long acting anti-anginal agents, digoxin, gout preventative agents, thyroid hormone replacement, lithium, and immunosuppressants. In states implementing overall caps, we evaluated the use of essential medications, symptomatic essential medications, and preventive essential medications. In states implementing brand caps, we evaluated the use of all branded medications and certain medication classes for which branded drugs and similar generics were available during the study period [19]; we included angiotensin-converting-enzyme (ACE) inhibitors, angiotensin receptor blockers (ARBs), calcium channel blockers (CCBs), statins, non-steroidal anti-inflammatory drugs (NSAIDs), proton-pump inhibitors (PPIs), selective serotonin reuptake inhibitors (SSRIs), and serotonin-norepinephrine reuptake inhibitors (SNRIs). For these classes combined, we evaluated the use of both branded and generic medications. For all outcomes, we examined the proportion of prescriptions and spending accounted for by each category of medications. Absolute numbers of prescriptions will change based on the number and composition of beneficiaries in a given time period and those data were not reliably available for our study period; accordingly, we used proportional outcomes.

### Analyses

We calculated outcomes for the quarter in which caps were implemented and six quarters before and after implementation (13 quarters), excluding quarters prior to 2001. The timeframe for each state’s data was standardized to the relative quarter in which the cap policy was initiated [20, 21]. The weighted average of outcomes in states without caps throughout the study period was used as a concurrent control series [20, 21].

We next developed segmented general linear models, adjusting for repeated observations, by using generalized estimating equations with an autoregressive correlation structure and a lag time of one quarter after initial cap implementation in that state. Models included terms indicating the temporal relationship of each quarter with cap implementation, including the immediate change (*level*) and ongoing change over time (*slope*). Terms for interaction between level and slope indicators and an indicator for whether states implemented or did not implement a cap were included to estimate the time-trend-adjusted effects of cap implementation.

There was considerable seasonal variation in the raw data, so we included sine and cosine terms to adjust for seasonality [22]. We also adjusted for level changes due to Medicare Part D implementation. Interaction terms between these parameters and the indicator for states implementing caps were included to adjust for between-group differences. In gathering policy data, we found notifications sent to providers 1–2 quarters before cap implementation, allowing providers to begin adjusting prescribing patterns. To more accurately estimate the impact of cap implementation, the quarter of implementation and the prior quarter were omitted from models. Complete model details can be found in Additional file 1 (online methods).

We used z-test results based on estimated β-coefficients and standard errors from the generalized linear models to determine statistical significance at a *P*-value <0.05. All analyses were conducted with SAS version 9.2 (SAS Institute Inc.). To approximate the impact of cap policy changes, we estimated the change in prescription utilization (prescriptions or expenditures) attributable to cap implementation based on segmented linear regression models. For each state implementing a cap, we calculated the utilization after cap implementation and the expected utilization if the cap had not been implemented (the counterfactual) based on these models. Next we calculated the difference between those two numbers and adjusted to annual estimates. We then conservatively adjusted all spending estimates downward by 20 % to account for the Medicaid drug rebate program [23, 24].

## Results

### Policies

Table 1 shows a complete list of all states’ prescription cap policies. We were unable to obtain policy data from Arizona and obtained only partial information for several states (see Additional file 1, online Table S2). Twenty-four states had prescription caps in place for Medicaid recipients at some point between 2001 and 2010. The number of states with caps increased from 12 in 2001 to 20 in 2010, with considerable increases in the use of caps on total medications *(overall caps)* and branded medications (*brand caps*) (Table 2). In 2001, there were no states with both overall and brand caps; by 2010, four states simultaneously used both policies. Overall cap levels ranged from 3–15 per month; brand cap levels ranged from 2–5 per month. The mean overall cap level (prescriptions per month) was 5.6 (*n* = 10, SD 2.6) in 2001 and 6.2 (*n* = 15, SD 3.5) in 2010. One state with an overall cap was excluded from calculations due to policy complexity (see Additional file 1, online results). In 2001, one state had a brand cap of 4 monthly prescriptions; in 2010, the mean brand cap level was 3.25 (*n* = 8, SD 1.3).

**Table 1:** Prescription cap policies by state, 2001-2010

State	Cap policy type	Policy start date	Policy end date	Overall prescription limit	Branded prescription limit
*Number of drugs (maximum number of exceptions) per member per month unless otherwise noted*					
Alabama	Brand	7/1/2004	12/31/2007	-	4 (10)
Alabama	Brand	1/1/2008	After 1/1/2011	-	5 (10)
Arkansas	Overall	Before 12/31/2000	After 1/1/2011	3 (3)	-
California	Overall	Before 12/31/2000	After 1/1/2011	6	-
Colorado	Overall	4/1/2003	6/2/2003	8	-
Delaware	Overall	1/1/2005	5/18/2009	15	-
Delaware	Overall	5/19/2009	After 1/1/2011	13	-
Florida	Brand	Before 12/31/2000	6/30/2005	-	4
Georgia	Overall	Before 12/31/2000	Oct-Dec 07^e^	5^f^	
Illinois	Brand	10/1/2005	After 1/1/2011	-	3
Kansas	Brand	4/1/2003	After 1/1/2011	-	5
Kentucky	Brand	4/19/2005	2/28/2006	-	3
Kentucky	Both	3/1/2006	After 1/1/2011	4	3
Louisiana	Overall	3/3/2003	4/30/2009	8	-
Louisiana	Overall	5/1/2009	11/30/2010	5	-
Louisiana	Overall	12/1/2010	After 1/1/2011	4	-
Maine^a^	Brand	11/2/2004^c^	6/30/2007	-	5
Maine	Brand	7/1/2007	After 1/1/2011	-	4
Mississippi	Overall	Before 12/31/2000	5/30/2002	10	-
Mississippi	Overall	6/1/2002	6/30/2005	5 (1)	-
Mississippi	Both	7/1/2005	After 1/1/2011	5	2
New York	Overall	Before 12/31/2000	8/31/2010	40/43 annually^g^	-
New York	Overall	9/1/2010^d^	After 1/1/2011	Clinical^h^	-
North Carolina	Overall	Before 12/31/2000	5/30/2006	6	-
North Carolina	Overall	6/1/2006	After 1/1/2011	8 (3)	-
Oklahoma	Overall	Before 12/31/2000	12/31/2003	3	-
Oklahoma	Both	1/1/2004	12/31/2009	6	3
Oklahoma	Both	1/1/2010	After 1/1/2011	6	2
Oregon	Overall	2/1/2004	After 1/1/2011	15^i^	-
Pennsylvania^a^	Overall	Before 12/31/2000	After 1/1/2011	6	-
South Carolina	Overall	Before 12/31/2000	7/19/2010	4 (6)	-
South Carolina	Overall	7/20/2010	After 1/1/2011	4 (4)	-
Tennessee	Both	8/1/2005	After 1/1/2011	5	2
Texas	Overall	Before 12/31/2000	After 1/1/2011	3	-
Utah^b^	Overall	1/1/2002	Between 2005-2010^e^	7	-
Utah^a,b^	Overall	7/1/2002	After 1/1/2011	7	-
Washington	Brand	2/1/2002	6/30/2007	-	4
West Virginia	Overall	Before 12/31/2000	7/14/2002	10	-
West Virginia^a^	Overall	March 2007^c^	After 1/1/2011	4	-

^a^ Policy affecting limited groups of recipients (see Text, Additional file 1 Digital Content 1, results)

^b^ Utah started a new program called non-traditional Medicaid (NTM) on this date with the cap in effect. The cap for traditional Medicaid was removed between 2005 and January 2010 but remained in place for NTM (see Text, Additional file 1 Digital Content 1, results)

^c^ Policy implemented gradually on this date

^d^ Policy gradually implemented starting in the second half of 2008 but was not enforced until 9/1/2010

^e^ The exact date of policy change was unclear based on available information (see Table 2, Additional file 1 Digital Content 2)

^f^ Recipients below age 21 limited to 6 prescriptions per month

^g^ Recipients <21 or ≥65, certified blind or disabled, or single caretaker of a child under 18 are limited to 40 prescriptions annually; all other recipients limited to 43 prescription annually (see Text, Additional file 1 Digital Content 1, results)

^h^ Annual prescription limits based on patient clinical information (see Text, Additional file 1 Digital Content 1, results)

^i^ May impose limitations on clients with prescriptions for more than 15 unique drugs in a 6 month period

**Table 2:** Medicaid prescription cap policies 2001 & 2010^a^

	2001		2010	
	States	Average Cap Level (range)	States	Average Cap Level (range)
Cap	12		20	
Overall only^b^	11	5.6 (3–10)	12	6.2 (3–15)
Brand only	1	4 (−)	4	3.25 (2–5)
Both	0		4^c^	
No cap	36		30	

^a^ Cap policies for three states in 2001 and one state in 2010 were not identified (see Table 2, Additional file 1 Digital Content 2)

^b^ One state in 2001 and three in 2010 had overall caps affecting limited groups of Medicaid recipients (see Text, Additional file 1 Content 1, results)

^c^ States with both cap types separately included in overall and brand average calculations

From 2001 to 2010, 15 states implemented 17 caps (8 overall, 9 brand) and six states removed caps (4 overall, 2 brand), though one state removed that cap for only some recipients (see Additional file 1, online results). Four states both implemented and removed prescription caps (3 overall, 1 brand). Eight states changed cap levels 11 times (9 overall, 2 brand), seven of which made coverage more restrictive for recipients. The impact of one change in overall cap levels could not be categorized due to policy complexity (see Additional file 1, online results). For information on unique cap policies and policy exceptions, see Additional file 1 (online results).

### Overall cap implementation

Essential medications accounted for a greater proportion of prescriptions (7.7 %, 95 % CI, 4.7 %-10.7 %, *p* < 0.001) and a slightly greater proportion of expenditures (4.0 %, 95 % CI, −0.25 %-8.2 %, *p* = 0.065) in states without caps as compared to states implementing overall caps (Table 3). After overall cap implementation, there was a 0.13 % (95 % CI, 0.05 %-0.21 %, *p* < 0.001) quarterly decrease (*slope effect*) in the proportion of prescriptions for essential medications, equivalent to 0.52 % per year. The change in slope for expenditures and immediate changes (*level effects*) for both comparisons were not significant (all, *p* > 0.10).

**Table 3:** Impact of overall and brand cap implementation on prescription utilization

Outcome	Impact of cap implementation (% change)		95 % Confidence interval	
OVERALL CAP IMPLEMENTATION				
*Essential medications*				
Prescriptions	Change in level	−0.28	−0.75	0.19
	Change in slope	−0.13**	−0.21	−0.05
Expenditures	Change in level	−0.28	−1.52	0.97
	Change in slope	−0.17	−0.66	0.32
*Preventive essential medications*				
Prescriptions	Change in level	−0.47	−1.05	0.1
	Change in slope	−0.28**	−0.46	−0.11
Expenditures	Change in level	−0.44	−1.35	0.46
	Change in slope	−0.30**	−0.43	−0.17
*Symptomatic essential medications*				
Prescriptions	Change in level	0.19**	0.07	0.31
	Change in slope	0.14	−0.05	0.34
Expenditures	Change in level	0.15	−0.31	0.61
	Change in slope	0.11	−0.49	0.71
BRAND CAP IMPLEMENTATION				
*All brand medications*				
Prescriptions	Change in level	−2.29*	−4.16	−0.42
	Change in slope	−0.02	−0.23	0.18
Expenditures	Change in level	−1.26*	−2.36	−0.16
	Change in slope	0.08	−0.19	0.35
*Brand medications in classes with generic replacements* ^*a*^				
Prescriptions	Change in level	−0.74**	−1.23	−0.25
	Change in slope	0.02	−0.22	0.26
Expenditures	Change in level	−1.27**	−2.01	−0.53
	Change in slope	0.05	−0.29	0.40
*Generic medication in classes with generic replacements* ^*a*^				
Prescriptions	Change in level	0.79**	0.20	1.38
	Change in slope	−0.08	−0.19	0.03
Expenditures	Change in level	0.60	−0.12	1.31
	Change in slope	−0.03	−0.13	0.08

Interrupted time-series analyses modeling the impact of cap policy implementation on the proportion of prescriptions for selected groups of medications, controlling for Medicare Part D implementation and season. Medication use in states without prescription caps was used as a control. “Level” refers to the immediate impact of cap policy implementation on proportion of medication use. “Slope” refers to the subsequent rate of change *per calendar quarter* in proportion of use resulting from the cap policy. Complete model parameters can be found in Additional file 1. *p < 0.05; **p < 0.01

^a^ Selected classes include: ACE-inhibitors, ARBs, CCBs, statins, NSAIDs, PPIs, SSRIs, and SNRIs

**Fig. 1:**
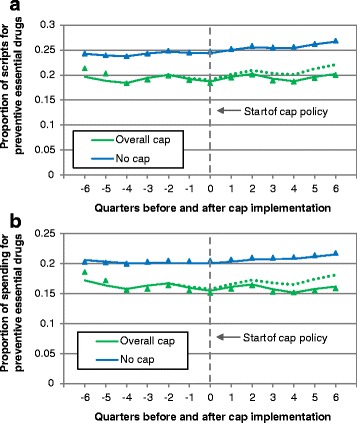
Proportion of prescriptions (**a**) and spending (**b**) accounted for by preventive essential drugs before and after implementation of overall cap policies. Triangles and squares represent measured proportion of utilization. Solid lines represent predicted utilization based on models. The dotted line represents predicted utilization if overall cap policies had not been implemented (the counterfactual). Time is measured in calendar quarters relative to policy implementation. The weighted average of medication use in states without prescription caps throughout the study period was used as a control. The timeframe for the control data was standardized relative to the quarter in which the cap policy was initiated in the intervention state

For preventive essential medications, there was a 0.28 % (95 % CI, 0.11 %-0.46 %, *p* = 0.001) quarterly slope decrease equivalent to 1.12 % per year in the proportion of prescriptions and a 0.30 % (95 % CI, 0.17 %-0.43 %, *p* < 0.001) decrease equivalent to 1.20 % per year in the proportion of spending after overall cap implementation (Fig. 1, Table 3); level changes for both comparisons were not significant (all, *p* > 0.10). For symptomatic essential medications, there was a 0.19 % (95 % CI, 0.07 %-0.31 %, *p* = 0.002) level increase in the proportion of prescriptions; however, the level change for expenditures and slope changes for both comparisons were not significant (all, *p* > 0.10). In the three states implementing overall caps, the decreased use of preventive essential medications attributable to cap implementation was 246,000 prescriptions (95 % CI, 156,000-341,000) and $12.2 million (95 % CI, $8.79-$15.5 million) annually.

### Brand cap implementation

Branded drugs accounted for approximately half of prescriptions but over 80 % of expenditures (see additional file 1, online Figure S1). Though the proportion of branded prescriptions decreased significantly by 0.59 % (95 % CI, 0.42 %-0.77 %, *p* < 0.001) per quarter equivalent to 2.36 % per year, branded expenditures did not significantly change (*p* > 0.10). Brand cap implementation led to a level decrease of 2.29 % (95 % CI, 0.42 %-4.16 %, *p* = 0.016) in the proportion of branded prescriptions and 1.26 % (95 % CI, 0.16 %-2.36 %, *p* = 0.025) in the proportion of branded expenditures; changes in slope were not significant (all, *p* > 0.10). In the six states examined, brand cap implementation was associated with a decrease of 1.53 million prescriptions (95 % CI 305,000-2.75 million) and $30.8 million (95 % CI −620,000-62.1 million).

**Fig. 2:**
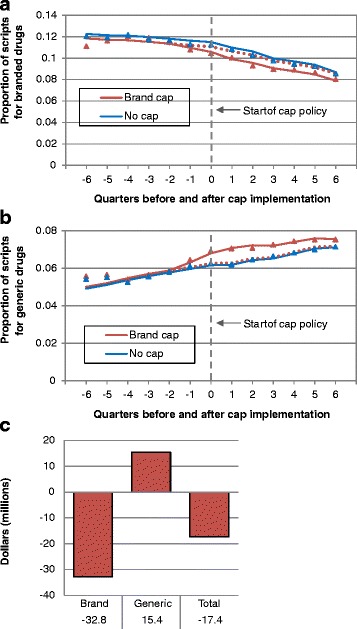
Proportion of branded (**a**) and generic (**b**) prescriptions and estimated spending changes for classes of drugs with generic replacements before and after implementation of brand cap policies. Selected classes include: ACE-inhibitors, ARBs, CCBs, statins, NSAIDs, PPIs, SSRIs, and SNRIs. **a**, **b** Triangles and squares represent measured proportion of utilization. Solid lines represent predicted utilization based on models. The dotted line represents predicted utilization if brand cap policies had not been implemented (the counterfactual). Time is measured in calendar quarters relative to policy implementation. The weighted average of medication use in states without prescription caps throughout the study period was used as a control. The timeframe for the control data was standardized relative to the quarter in which the cap policy was initiated in the intervention state. **c** Estimated spending changes for medication classes with generic replacements due to brand cap implementation, for brand drugs, generic drugs, and all drugs

Among medication classes with available generic replacements (ACE-inhibitors, ARBs, CCBs, statins, NSAIDs, PPIs, SSRIs, and SNRIs), brand cap implementation led to a level decrease of 0.74 % (95 % CI, 0.25 %-1.23 %, *p* = 0.003) in the proportion of branded prescriptions and a contrasting level increase of 0.79 % (95 % CI, 0.20 %-1.38 %, *p* = 0.009) for generic prescriptions (Fig. 2; Table 3). While spending on these branded drugs decreased significantly by 1.27 % (95 % CI, 0.53 %-2.01 %, *p* < 0.001), increased spending on generics was only marginally significant (0.60 %; 95 % CI, −0.12 %-1.31 %, *p* = 0.10). Changes in slope were not significant for all comparisons (all, *p* > 0.10). Among these medication classes, the decreased use of branded prescriptions attributable to cap implementation was 457,000 (95 % CI, 136,000-778,000) and the increased use of generic prescriptions was 397,000 (95 % CI, 11,300-783,000). The decreased spending on branded drugs attributable to brand cap implementation was $32.8 million (95 %, CI 11.8-53.8 million), while the increase in spending on generics was $15.4 million (95 %, CI −5.02-35.9 million), resulting in estimated savings of $17.4 million (Fig. 2c). Unadjusted outcomes for all comparisons and complete parameter estimates for all models can be found in Additional file 1 (online Tables S3 and S4, respectively).

## Discussion

The number of state Medicaid programs with prescription caps rose considerably from 12 in 2001 to 20 by the end of 2010, with a sharp rise in brand caps, a policy used by only one state in 2001. The combined Medicaid enrollment in the 20 states with caps in 2010 was 29.8 million, 58 % of total Medicaid enrollees [1]. The restrictiveness of recent cap policies varied greatly, particularly for overall caps, which ranged from 3–15 prescriptions per month.

Overall cap implementation led to slightly decreased use of preventive essential medications but not essential medications that provide symptomatic benefit. This suggests that, when faced with caps, some patients are willing to forgo medications with little symptomatic benefit to maintain the use of those that provide short-term benefit. Preventive essential medications include highly effective drugs that decrease morbidity and mortality for common conditions such as hypertension and diabetes, which affect tens of millions of Medicaid recipients [25–29]. These are also medications for which patients are often poorly adherent [30, 31]. A policy that selectively decreases the usage of such medications may not be cost-effective or even cost saving when clinical consequences are taken into account [32].

These findings are particularly concerning in the context of prior evidence demonstrating the harms of overall caps [4–6]. In 1981, New Hampshire Medicaid implemented a three drug per month cap that led to decreased use of essential medications and increased nursing homes admissions [4, 5]. As the number of effective medications has dramatically increased over the past several decades, the potential for overall caps to worsen health outcomes warrants concern [25, 26].

When interpreting our results, it is important to keep in mind the rationale for prescription cap policies. Such policies are generally enacted to address the concern that patients receiving support from a public insurance program are using public funds to purchase medications that are in some way undesirable (e.g., medications for elective indications or those that are overly expensive). Cap policies implicitly assume that patients will review the list of medications they have been taking, choose those that are most important for their overall health, and forego less important medications. In practice, however, patients receive and fill prescriptions intermittently, dependent mostly on when clinical visits occur. Accordingly, patients are unlikely to consider their entire list of medications, but instead are more likely to confront prescription caps unpredictably when they arrive at the pharmacy to pick up a prescription. Caps implemented in this manner may be just as likely to prevent the filling of important or relatively inexpensive prescriptions as to reduce the overuse of less important treatments. Our findings indicate that this type of policy tool may not have the desired effect at the patient level.

Given how cap policies are implemented, it would be challenging for physicians or pharmacists to consistently help patients navigate these policies. Typically, caps take effect for each individual patent at the pharmacy level. Prescriptions exceeding the monthly cap level are not reimbursed and patients are required to pay full price for these medications or forego the prescriptions. Most individuals in the United States fill prescriptions at freestanding pharmacies not associated with outpatient clinics. Therefore, it is unlikely that a physician writing a new prescription would be aware at that moment of whether a prescription exceeded an individual’s limit for each month. It is conceivable that a primary care physician could attempt to track each Medicaid patient’s filled prescriptions relative to the state cap; however, this would require careful coordination between patient, physician, and pharmacy. This might be particularly challenging for Medicaid recipients, who have lower levels of education and health literacy. If a patient receives prescriptions from multiple providers, this would also complicate a physician’s ability to help patients manage caps. Pharmacists might be able to assist patients when deciding which prescriptions to fill, though this role would primarily be limited to patients filling multiple simultaneous prescriptions. Prior research has attempted to quantify the time and cost impact of physician interaction with health plans, but to our knowledge, there are no specific evaluations of the processes that would be required for physicians to help patients deal with cap policies [33].

Whether overall caps produce economic savings is unknown. We found that overall caps generated relative savings for some groups of medications. The 1981 New Hampshire cap generated modest savings of ~ $400,000 compared to the subsequent $1 copayment policy; however, a conservative estimate of the costs due to increased nursing home admissions approximately equaled those savings [4, 5]. Medicare Choice recipients in California with annual drug benefits capped at $1000 had total medical costs that were nearly identical to those whose drug benefits were not limited, as well as higher rates of emergency visits, hospitalizations and death, although it is not possible to rule out case-mix differences [34]. None of these prior analyses took into account the costs associated with clinician or office staff time required to address policies like these, which, as noted, may be considerable [33].

Caps on branded drugs represent a newer policy approach that has not previously been studied. Brand cap implementation led to significantly decreased use of brand medications. For medication classes where therapeutic substitution with generics was possible, the decrease in branded prescriptions approximately equaled the increase in generics. In contrast, the estimated net savings among these medication classes was $17.4 million due to the lower cost of generics. Prior research has demonstrated considerable economic waste due to branded medication use when clinically equivalent or superior generics are available [35–37]. For medication classes with available generic equivalents, thoughtfully designed brand cap policies might effectively shift use from branded to generic medications and generate savings. We were not able to study branded medications without available generic replacements due to inadequate sample size.

Our study has several limitations. Because the data are aggregate, we cannot examine patient-level prescription use; however, our analyses demonstrate decreases in medication use after cap implementation, findings consistent with prior patient-level studies [4–6, 34, 38]. Additionally, our approach only allowed for examination of newly implemented caps. We analyzed the impact of overall cap implementation in three states, though 16 employed overall caps at the end of 2010. When compared to other overall caps, these three policies were among the least restrictive (Table 1). More restrictive policies already in place might have a considerably greater impact on prescription use. The limited number of states with new policies in the critical time window and the use of aggregate quarterly data meant that our analytic sample included a relatively small number of observations, limiting the degrees of freedom and preventing us from including additional variables in our models. Future analyses with access to more years of data or individual-level data might allow these factors to be explored.

Our control group consisted of states without caps. It is possible that those states systematically differed from states implementing caps. States without caps had higher baseline use of essential medications and may have differed in their patient populations or other pharmaceutical cost-control policies [39]. Nonetheless, as control states were numerous and diverse, they are probably representative of underlying trends in prescription utilization and provide a suitable control for co-interventions or events at the national level (e.g., recall of a specific drug) that would impact prescription use.

We cannot determine if prescription utilization decreased due to changes in physician or patient behavior. It is unlikely that the changes we observed can be explained by out-of-pocket payment (e.g., through retail pharmacy chain “$4 generics” programs) in this indigent population, or by short-term use of free samples [7, 8, 40]. We also cannot control for prescription usage among those not affected by caps, however, the inclusion of prescriptions used by these groups would likely weaken any associations. Other approaches, such as preferred drug lists or more restrictive policies already in place, might also impact prescription use.

The vulnerable nature of those insured by Medicaid also demands special policy consideration. Medicaid primarily provides coverage to children, pregnant women, the disabled, and the elderly. Underserved racial and ethnic minorities are also disproportionately served by Medicaid. These populations often have reduced capacity to navigate the healthcare system, increased disease burden, limited access to healthcare, and worse health outcomes [29, 41–43]. Indigent Medicaid recipients also have limited disposable income to buy prescriptions not covered by their insurance and are therefore particularly likely to be harmed by policies limiting access to effective medications. Within the Medicaid population, caps may disproportionately affect the disabled and the elderly, leading to increased health disparities among those socially disadvantaged groups.

## Conclusion

In summary, we found a substantial increase in the use of Medicaid prescription caps, including limits on the overall number of prescriptions and limits on the number of branded prescriptions. Overall cap implementation led to decreased use of preventative essential medications but not symptomatic essential medications, suggesting that patients assign higher priority to agents providing symptomatic benefit when faced with reimbursement limits. Among medication classes with available generic replacements, brand cap implementation shifted usage from branded drugs to generics without decreased use, resulting in considerable savings.

A substantial proportion of Medicaid recipients are affected by prescription caps, a number which is likely to grow in the coming years. The recent recession and subsequent recovery have increased Medicaid enrollment and decreased state revenues [2, 3, 44]. Additionally, the ACA has expanded Medicaid eligibility to include people with household incomes at or below 133 % of the federal poverty level; this may increase Medicaid enrollment by 16–22 million by 2019, though these estimates do not account for the Supreme Court’s decision changing the Medicaid expansion into an optional program [45–47]. Thus, for the foreseeable future, Medicaid will be the insurance provider for a growing proportion of individuals in the United States under increasing financial constraints. Given these challenging circumstances, states are likely to consider additional prescription cost-control measures. Determining the clinical and economic impact of different policies, such as prescription caps, will help guide sound pharmaceutical policy.

## Additional file

Additional file 1:
**Online methods.** Online results. Online **Table S1.** Sources of policy information organized by state. Online **Table S2.** Missing state policy information. Online **Table S3.** Proportion of prescription usage for medications before and after cap implementation. Online **Table S4.** Model parameter estimates. Online **Figure S1.** Proportion of prescriptions and spending accounted for by brand drugs before and after implementation of brand cap policies. (DOCX 165 kb)

## Abbreviations

ACA: Patient protection and affordable care act
CMS: Center for medicare and medicaid services
NDC: National drug code
NSAID: Non-steroidal anti-inflammatory drug
ARB: Angiotensin receptor blocker
CCB: Calcium channel blocker
PPIs: Proton-pump inhibitors
SSRI: Selective serotonin reuptake inhibitor
SNRI: Serotonin-norepinephrine reuptake inhibitor
