# Supplementary material for: Medicaid prescription limits: policy trends and comparative impact on utilization
Source: BMC Health Serv Res. 2016 Jan 15;16:15. doi: 10.1186/s12913-016-1258-0 (PMC4714442; doi:10.1186/s12913-016-1258-0)
Supplement: Additional file 1: — Online methods. Online results. Online Table S1. Sources of policy information organized by state. Online Table S2. Missing state policy information. Online Table S3. Proportion of prescription usage for medications before and after cap implementation. Online Table S4. Model parameter estimates. Online Figure S1. Proportion of prescriptions and spending accounted for by brand drugs before and after implementation of brand cap policies. (DOCX 165 kb) [file 12913_2016_1258_MOESM1_ESM.docx]

**Medicaid prescription limits: policy trends and comparative impact on utilization**

***Online appendix***

**Contents**

1. Online methods
2. Online results
3. Online table 1. Sources of policy information organized by state
4. Online table 2. Missing state policy information
5. Online table 3. Proportion of prescription usage for medications before and after cap implementation
6. Online table 4. Model parameter estimates
7. Online figure 1. Proportion of prescriptions and spending accounted for by brand drugs before and after implementation of brand cap policies

**Online methods**

**Quality control**

For state prescription utilization data, we performed quality checks to identify erroneous entries; we excluded quarters for which total prescriptions or expenditures differed by greater than 50% from both the prior and subsequent quarter and imputed estimates based on adjacent values. Values for 14 of 734 possible quarters (1.9%; 3 from states implementing caps, 11 from control states) were identified using this approach. Additionally, data for three quarters from control states were not available and were imputed based on adjacent values. Six states without caps were excluded from the control group due to consecutive censored quarters or due to unexplained and implausible raw data variations.

**Selection of cap policy interventions**

We evaluated policies in states that implemented overall caps or brand caps as a sole intervention. We excluded states that implemented a second cap (e.g. a state with a brand cap implementing an overall cap), simultaneously implemented brand and overall caps, or simultaneously implemented caps and copayments. We also excluded states that implemented caps applying to a limited group of recipients (e.g. those not meeting federal Medicaid eligibility criteria) or caps that were in place for less than 6 quarters.

Of note, one state that implemented a brand cap (ME) simultaneously increased copayments for all prescriptions by $0.50 and previously had a brand cap in place for a limited group of recipients. For two states that implemented caps during the first quarter of 2002 (WA, UT), we included only 4 quarters of data prior to the policy change. Additionally, one state that implemented a brand cap (KY) subsequently implemented an overall cap three quarters after brand cap implementation.

**Explanation of segmented linear regression models**

*Proportion of utilization = β_0_ + β_1_*Time + β_2_*Cap_state + β_3_*Post_policy_indicator + β_4_*Post_policy_time + β_5_*Part_D + β_6_*sin(time) + β_7_*cos(time) + β_8_*Part_D*Cap_state + β_9_*sin(time)*Cap_state + β_10_*cos(time)*Cap_state +* ***β_11_*Post_policy_indicator*Cap_state +*** ***β_12_*Post_policy_time_*Cap_state*** *+ ε*

Time: variable to indicate time in quarters relative to policy implementation (0 to 13)

Cap_state: variable to indicate states implementing caps versus control states

Post_policy_indicator: variable to indicate quarters occurring after cap implementation (level change)

Post_policy_time: variable to indicate time trend after cap implementation (slope change)

Part_D: variable to indicate quarters occurring after Part D implementation

Sin(time) and cos(time): variables to adjust for seasonality

Part_D*cap_state: interaction term, adjusts for differences in level changes after Part D implementation between states implementing caps and those without caps

Sin(time)*cap_state and cos(time)*cap_state: interaction terms, adjust for differences in seasonality between states implementing caps and those without caps

**Post_policy_indicator*cap_state:** interaction term, measures the effect of level change due to cap implementation, adjusting for states without caps

**Post_policy_time*cap_state:** interaction term, measures the effect of slope change due to cap implementation, adjusting for states without caps

**Online results**

**Unique and illustrative prescription cap policies and events**

During the study period, three states (DE, OR, UT) implemented "soft" prescription caps for Medicaid enrollees. In these states, exceeding the cap triggered a drug utilization review. In Oregon, override authority lay with the state DUR board; in Utah, the recipient’s physician had final override authority. Prior to implementing this “soft” cap, Utah intended to implement a firm cap; however, the policy was changed immediately prior to implementation due to the concerns of advocacy groups and clients. Colorado implemented an overall cap limiting Medicaid recipients to 8 prescriptions per month in 2003, which was removed two months later. In New York, recipients were limited to 40 or 43 prescriptions annually based on age and enrollment status at the beginning of the study period. In 2010, limits were changed and were subsequently based on individual patient clinical characteristics. Accordingly, New York was not included in calculations of average of cap level and changes in cap level.

Four states (ME, PA, UT, WV) enforced prescription caps for only some recipients during the study period. Generally these caps were applied to patients based on eligibility. The overall cap in Utah described above was initially implemented for all Medicaid recipients; later that year, Utah started non-traditional Medicaid (NTM), a program which provides services to adults who qualify because they have a dependent child on Medicaid, in which the cap was also enforced. The cap was later removed for traditional Medicaid recipients and remained in place for recipients enrolled in NTM. In Pennsylvania, patients in the Medical Assistance (MA) program, which covers some populations that do not meet federal criteria for Medicaid eligibility, were limited to six prescriptions per month. Maine initially implemented a brand cap of five drugs per month for recipients in nursing homes and other supervised settings as well as non-categorically eligible single, childless adults; the state subsequently implemented a four drug per month brand cap for all Medicaid recipients. In West Virginia beginning in 2007, certain groups of federally eligible Medicaid recipients who did not sign and uphold a “Medicaid Member Agreement” in which they pledged to fulfill certain responsibilities, such as keeping their appointments and taking their medications, were limited to four prescriptions per month in addition to multiple other restrictions.

**Unique and illustrative prescription cap policy exceptions**

We broadly describe policy exceptions here; for detailed information about cap policy exceptions, please contact the author. For both overall and brand caps, the extent of excepted medications varied greatly. Several states with both brand and overall caps did not have any automatically excepted medications, while other states had lists of over 20 classes of automatically excepted medications. For example, beginning in 2005 Mississippi had an overall cap of five drugs per month and a brand cap of two drugs per month from which no medications were automatically excepted. Nine classes of medications were automatically excepted from the five drug brand cap that Kansas implemented in 2003. Frequently excepted classes of medications included antipsychotics, anti-retrovirals, anti-neoplastics, and contraceptives. Most, but not all states had procedures for overriding caps for medically necessary medications, frequently operationalized through a prior authorization process. Several states also had a maximum number of drug exceptions a patient could receive above the baseline cap (**table 2**). Children and those in long-term care facilities were the recipients most frequently excepted from prescription caps, though in several states no recipients were automatically excepted. Whether prescription caps applied to Medicaid managed care enrollees varied by state.

The complexity of both the process for obtaining exceptions and determining which medications were excepted from caps varied greatly by state. Some states enforced simple policies in which all medications in certain classes were excepted. In California, anti-retrovirals, anti-neoplastics, and family planning medications and supplies were automatically excepted from the six drug per month overall cap. In some states, medication exceptions were considerably more complex. In Tennessee, three mechanisms existed to override a five drug overall cap and two drug brand cap. Drugs from 20 classes in the “auto-exemption list” were automatically excepted from caps without necessitating authorization. The “dose titration list” included 11 classes of drugs that require frequent titration for which a second prescription in one month did not count toward the prescription limit. Finally, the “prescriber attestation list” included “approximately 500 medications in 20 drug categories” for which physicians could request prior authorization if the medication was necessary to prevent serious health consequences. This final process required both a phone call and subsequent fax to the state pharmacy benefits manager. The details of these policies can be found at the Tennessee pharmacy provider website (<http://www.tn.gov/tenncare/pro-pharmacy.html>).

**Online table 1. Sources of policy information organized by state**

| **State** | **Source** | **Section** | **Web Address** |
| --- | --- | --- | --- |
| Alabama | State Medicaid website | Current and archived provider manuals | http://medicaid.alabama.gov/CONTENT/6.0_Providers/6.7_Manuals.aspx |
|  | State Medicaid website | News and notices | http://medicaid.alabama.gov/CONTENT/6.0_Providers/6.8_News_and_Notices.aspx |
| Alaska | State Medicaid website | Pharmacy billing manual | http://medicaidalaska.com/providers/Billing1.shtml |
|  | State Medicaid website | Pharmacy notices | http://medicaidalaska.com/providers/rx/default.shtml |
|  | State Medicaid website | Provider updates | http://medicaidalaska.com/providers/provupdates.shtml |
| Arizona | N/A |  |  |
| Arkansas | State Medicaid website | Pharmacy provider documents | https://www.medicaid.state.ar.us/InternetSolution/Provider/docs/pharmacy.aspx |
| California | State Medicaid website | Provider manuals | http://files.medi-cal.ca.gov/pubsdoco/manuals_menu.asp |
| Colorado | State Medicaid website | Provider manuals | http://www.colorado.gov/cs/Satellite/HCPF/HCPF/1201542697088 |
|  | State Medicaid website | Provider bulletins | http://www.colorado.gov/cs/Satellite/HCPF/HCPF/1246972411343 |
| Connecticut | State Medicaid website | Provider publications | https://www.ctdssmap.com/CTPortal/Information/Publications/tabId/40/Default.aspx |
| Delaware | State Medicaid website | Pharmacy manual | http://www.dmap.state.de.us/information/pharmacy.html |
|  | State Medicaid website | Drug utilization review board minutes | http://www.dmap.state.de.us/information/DURboardnotes.html |
|  | State Medicaid website | Provider bulletins | http://www.dmap.state.de.us/downloads/bulletinarchive.html |
|  | Direct contact with state Medicaid office |  |  |
| DC | State Medicaid website | Provider manuals |  |
|  | State Medicaid website | Provider bulletins/transmittals | https://www.dc-medicaid.com/dcwebportal/nonsecure/transmittals |
|  | Medicaid Pharmacy Services website | Provider manual | http://www.dcpbm.com/provider_manuals.html |
| Florida | State Medicaid website | Provider handbooks | http://portal.flmmis.com/FLPublic/Provider_ProviderSupport/Provider_ProviderSupport_ProviderHandbooks/tabId/42/Default.aspx |
|  | State Medicaid website | Provider notices | http://portal.flmmis.com/FLPublic/Provider_ProviderSupport/Provider_ProviderSupport_ProviderNotices/tabId/41/Default.aspx |
|  | State Medicaid website | Provider bulletins | http://portal.flmmis.com/FLPublic/Provider_ProviderSupport/Provider_ProviderSupport_ProviderBulletins/tabId/40/Default.aspx |
|  | Direct contact with state Medicaid office |  |  |

**Online table 1 (continued). Sources of policy information organized by state**

| **State** | **Source** | **Section** | **Web Address** |
| --- | --- | --- | --- |
| Georgia | State Medicaid website | Provider manuals | https://www.mmis.georgia.gov/portal/PubAccess.Provider%20Information/Provider%20Manuals/tabId/54/Default.aspx |
|  | Direct contact with state Medicaid office |  |  |
| Hawaii | State Medicaid website | Provider manuals | http://www.med-quest.us/providers/ProviderManual.html |
|  | State Medicaid website | Provider bulletins | http://www.med-quest.us/providers/ProvidersMemos.html#ProvidersPDLmemos |
|  | Hawaii Prescription Benefits | Pharmacy memos | http://www.himed-questffs.org/ |
| Idaho | Pharmacy program website | Pharmacy manual | https://idaho.fhsc.com/default.asp |
|  | State Medicaid website | Information releases | http://healthandwelfare.idaho.gov/Providers/PrescriptionDrugProviders/tabid/205/Default.aspx |
|  | State Medicaid website | Newsletters | http://healthandwelfare.idaho.gov/Providers/MedicaidProviders/MedicAideNewsletter/tabid/267/Default.aspx |
| Illinois | State Medicaid website | Provider handbooks | http://www.hfs.illinois.gov/handbooks/chapter200.html |
|  | State Medicaid website | Pharmacy provider notices | http://www.hfs.illinois.gov/pharmacies/ |
| Indiana | State Medicaid website | Manuals | http://provider.indianamedicaid.com/general-provider-services/manuals.aspx |
|  | State Medicaid website | News, Bulletins, and Banner Pages | http://provider.indianamedicaid.com/news,-bulletins,-and-banners.aspx |
| Iowa | State Medicaid website | Provider manuals | http://www.dhs.state.ia.us/policyanalysis/PolicyManualPages/MedProvider.htm#All%20Provider%20Chapters |
|  | State Medicaid website | Provider bulletin | http://www.ime.state.ia.us/Providers/Bulletins.html |
|  | State legislature website | Administrative bulletins | http://www.legis.state.ia.us/Rules/Current/Bulletin/ |
| Kansas | State Medicaid website | Current and archived provider manuals | https://www.kmap-state-ks.us/public/providermanuals.asp |
| Kentucky | State Medicaid website | Provider letters | http://chfs.ky.gov/dms/2004.htm |
|  | State Medicaid website | Pharmacy resources | http://chfs.ky.gov/dms/Pharmacy.htm |
|  | Kentucky pharmacy home | Pharmacy provider billing manual | https://kentucky.magellanmedicaid.com/Providers/Manuals.asp |
|  | Kentucky pharmacy home | Pharmacy provider notices | https://kentucky.magellanmedicaid.com/Providers/HistoricalBulletins.asp |
|  | State Medicaid website | Member handbook | http://chfs.ky.gov/dms/member+information.htm |

**Online table 1 (continued). Sources of policy information organized by state**

| **State** | **Source** | **Section** | **Web Address** |
| --- | --- | --- | --- |
| Louisiana | State Medicaid website | Provider manuals | http://www.lamedicaid.com/provweb1/Providermanuals/Intro_Page.aspx |
|  | State Medicaid website | Provider forms | http://www.lamedicaid.com/provweb1/forms/forms.htm |
| Maine | State website | Provider manuals | http://www.maine.gov/sos/cec/rules/10/ch101.htm |
|  | Pharmacy program website | General pharmacy info; memos and mailings | http://www.mainecarepdl.org/index.pl/genpharmfiles |
|  | State website | Weekly Rule-making Notices | http://www.maine.gov/sos/cec/rules/notices.html |
| Maryland | State pharmacy website | Provider information | http://mmcp.dhmh.maryland.gov/pap/SitePages/paphome.aspx |
|  | State Medicaid pharmacy website | Newsletters | http://www.marylandmedicaidpharmacyinformation.com/Newsletters.htm |
| Massachusetts | State Medicaid website | Pharmacy manual | http://www.mass.gov/eohhs/provider/insurance/masshealth/pharmacy/masshealth-pharmacy-regs.html |
|  | State Medicaid website | Provider bulletins | http://www.mass.gov/eohhs/gov/laws-regs/masshealth/provider-library/provider-bulletins/ |
|  | State Medicaid website | Pharmacy facts | http://www.mass.gov/eohhs/provider/insurance/masshealth/pharmacy/facts/ |
| Michigan | State Medicaid website | Provider manual | http://www.michigan.gov/mdch/0,1607,7-132--87572--,00.html |
|  | State Medicaid website | Policy bulletins | http://www.michigan.gov/mdch/0,1607,7-132--87513--,00.html |
| Minnesota | State Medicaid website | Provider manuals | <http://www.dhs.state.mn.us/main/idcplg?IdcService=GET_DYNAMIC_CONVERSION&RevisionSelectionMethod=LatestReleased&dDocName=dhs16_157386> |
|  | State Medicaid website | Provider updates | http://www.dhs.state.mn.us/main/idcplg?IdcService=GET_DYNAMIC_CONVERSION&RevisionSelectionMethod=LatestReleased&dDocName=id_010495 |
|  | State Medicaid website | Pharmacy information | http://www.dhs.state.mn.us/main/idcplg?IdcService=GET_DYNAMIC_CONVERSION&RevisionSelectionMethod=LatestReleased&dDocName=id_009298 |
|  | State office of the revisor of statutes website | Rules and statutes | https://www.revisor.mn.gov/rules/ |

**Online table 1 (continued). Sources of policy information organized by state**

| **State** | **Source** | **Section** | **Web Address** |
| --- | --- | --- | --- |
| Mississippi | State Medicaid website | Provider policy manuals | <http://www.dhs.state.mn.us/main/idcplg?IdcService=GET_DYNAMIC_CONVERSION&RevisionSelectionMethod=LatestReleased&dDocName=dhs16_157386> |
|  | State Medicaid website | Provider bulletins | https://msmedicaid.acs-inc.com/msenvision/providerBulletins.do |
|  | Direct contact with state Medicaid office |  |  |
| Missouri | State Medicaid manuals website | Pharmacy manual | http://manuals.momed.com/manuals/ |
|  | State Medicaid website | Provider bulletins | http://www.dss.mo.gov/mhd/providers/pages/bulletins.htm |
| Montana | State Medicaid website | Pharmacy website: pharmacy manual | http://medicaidprovider.hhs.mt.gov/providerpages/providertype/19.shtml#manuals |
|  | State Medicaid website | Pharmacy website: notices and replacement Pages | http://medicaidprovider.hhs.mt.gov/providerpages/providertype/19.shtml#manuals |
|  | State Medicaid website | Medicaid Newsletters | http://medicaidprovider.hhs.mt.gov/providerpages/newsletters.shtml |
| Nebraska | State Medicaid website | Rules and Regulations | http://dhhs.ne.gov/medicaid/Pages/med_regs.aspx |
|  | State Medicaid pharmacy website | Pharmacy Claims Submission Manual | https://nebraska.fhsc.com/Providers/Manuals.asp |
|  | State Medicaid pharmacy website | Nebraska Medicaid General information: Fiscal Year 2006 | http://dhhs.ne.gov/Documents/MEDRPT06.pdf |
| Nevada | State Medicaid website | Medicaid Services Manuals and archives | http://dhcfp.state.nv.us/MSM%20Table%20of%20Contents.htm?Accept |
| New Hampshire | State Medicaid website | Pharmacy benefits management | http://www.dhhs.nh.gov/ombp/pharmacy/index.htm |
|  | State Medicaid provider services website | Provider bulletins | http://www.nhmedicaid.com/Downloads/bulletins.html |
|  | Medicaid administration website | Pharmacy provider manual | https://newhampshire.magellanmedicaid.com/portal/spring/main;jsessionid=pyr3PChGTyQvHC6wg115tCRyhQ5hxTSjnvbpdtKbGndnnyLN8n2m!335484054?execution=e1s2 |
| New Jersey | State Medicaid website | Newsletters and alerts | <https://www.njmmis.com/documentDownload.aspx?fileType=076B9D7D-96DC-4C8A-B9CE-BA46F47DDE1F> |
|  | Michie's legal resources | New Jersey legal code, pharmacy services | http://www.michie.com/newjersey/lpext.dll?f=templates&fn=main-h.htm&cp |
| New Mexico | State Medicaid website | Program policy manual | http://www.hsd.state.nm.us/mad/RPolicyManual.html |
|  | State register website | Cumulative website | http://www.nmcpr.state.nm.us/nmregister/Cumulative_index/cumulative_index.htm |

**Online table 1 (continued). Sources of policy information organized by state**

| **State** | **Source** | **Section** | **Web Address** |
| --- | --- | --- | --- |
| New York | State claims processing system website | Pharmacy manuals | https://www.emedny.org/ProviderManuals/Pharmacy/index.aspx |
|  | State claims processing system website | Pharmacy manual archive | https://www.emedny.org/ProviderManuals/Pharmacy/archive.aspx |
|  | State claims processing system website | ProDUR manual | https://www.emedny.org/ProviderManuals/Pharmacy/ProDUR-ECCA_Provider_Manual/index.aspx |
|  | State Medicaid website | Medicaid updates | http://www.health.ny.gov/health_care/medicaid/program/update/main.htm |
|  | State department of health website | Codes, rules and regulations | http://www.health.ny.gov/regulations/nycrr/title_18/ |
|  | Direct contact with state Medicaid office |  |  |
| North Carolina | State Medicaid website | Provider Manuals | http://www.ncdhhs.gov/dma/mp/index.htm |
|  | State Medicaid website | Medicaid bulletins | http://www.ncdhhs.gov/dma/bulletin/index.htm |
|  | State Medicaid website | Provider newsletters | http://www.ncdhhs.gov/dma/pharmnews/index.htm |
| North Dakota | State Medicaid website | Medicaid provider information, general providers and pharmacy manuals | http://www.nd.gov/dhs/services/medicalserv/medicaid/provider-all.html |
|  | State Legislative branch website | Budget committee on health care report | http://www.legis.nd.gov/assembly/58-2003/interim-info/final-reports/hcfinal.html |
| Ohio | State Medicaid website | Pharmacy Provider Manual: Policy and Procedure Guide | http://jfs.ohio.gov/ohp/bhpp/omdp/POS.stm |
|  | State department of job and family services website | Pharmacy manual | http://emanuals.odjfs.state.oh.us/emanuals/GetDocument.do?nodeId=%23node-id(695)&docId=Document(storage%3DREPOSITORY%2CdocID%3D%23node-id(2077947))&locSource=input&docLoc=%24REP_ROOT%24%23node-id(2077947)&version=8.0.0 |
|  | State department of job and family services website | Administrative code rules and archive | <http://emanuals.odjfs.state.oh.us/emanuals/GetTocDescendants.do?nodeId=%23node-id(693)&maxChildrenInLevel=100&version=8.0.0> |
|  | State department of job and family services website | Manual transmittal letters | http://www.odjfs.state.oh.us/lpc/mtl/index.asp#MAL |

**Online table 1 (continued). Sources of policy information organized by state**

| **State** | **Source** | **Section** | **Web Address** |
| --- | --- | --- | --- |
| Oklahoma | State Medicaid website | Policies and rules | http://www.okhca.org/xPolicy.aspx?id=734 |
|  | State Medicaid website | Pharmacy updates and archives | http://www.okhca.org/providers.aspx?id=1228 |
|  | Department of health services website | Policy transmittals | http://www.okdhs.org/library/policy/pt/ |
|  | Direct contact with state Medicaid office |  |  |
| Oregon | State Medicaid website | Pharmaceutical services rulebook history | http://www.dhs.state.or.us/policy/healthplan/history/pharmacy/main.html |
|  | State Medicaid website | General rulebook history | http://www.dhs.state.or.us/policy/healthplan/history/genrules/main.html |
|  | State Medicaid website | Transmittals | http://www.dhs.state.or.us/policy/healthplan/transmit/main.htm |
| Pennsylvania | State Medicaid website | Medical assistance regulations | http://www.dpw.state.pa.us/publications/forproviders/regulationshandbooksguidesandmanuals/medicalassistanceregulations/index.htm |
|  | State Medicaid website | Bulletins | http://services.dpw.state.pa.us/olddpw/bulletinsearch.aspx |
|  | State legal code | State legal code | http://www.pacode.com/ |
| Rhode Island | State Medicaid website | Pharmacy medical coverage policies | http://www.dhs.ri.gov/ForProvidersVendors/ServicesforProviders/ProviderManuals/Pharmacy/tabid/463/Default.aspx |
|  | State Medicaid website | Provider updates | http://www.dhs.ri.gov/ForProvidersVendors/MedicalAssistanceProviders/ProviderUpdates/tabid/167/Default.aspx |
| South Carolina | State Medicaid website | Pharmacy provider manual | http://www.scdhhs.gov/openpublic/ServiceProviders/ProviderManualsAll.asp?pType=Pharmacy |
|  | State Medicaid website | Press releases/bulletins | http://www2.scdhhs.gov/press-releases?page=1 |
|  | State Medicaid website | Provider newsletter | http://www.scdhhs.gov/Internet/pdf/Sept%2008%20Newsletter.pdf |
|  | State legislative audit council | Cost containment report | http://lac.sc.gov/LAC_Reports/2003/Pages/Medicaid.aspx |
| South Dakota | State Medicaid website | Provider manuals | http://dss.sd.gov/sdmedx/includes/providers/billingmanuals/index.aspx |
|  | State Medicaid website | Provider newsletters | http://dss.sd.gov/sdmedx/includes/providers/archive/newsLetters.aspx |
|  | State Medicaid website | Pharmacy provider information | http://dss.sd.gov/sdmedx/includes/providers/programinfo/pharmacy/index.aspx |
|  | State Medicaid website | Recipient handbook | http://dss.sd.gov/formspubs/docs/MEDSRVCS/RecipientHandbookAugust2011.pdf |

**Online table 1 (continued). Sources of policy information organized by state**

| **State** | **Source** | **Section** | **Web Address** |
| --- | --- | --- | --- |
| Tennessee | State Medicaid website | Pharmacy manual | http://www.tn.gov/tenncare/pro-pharmacy.html |
|  | State Medicaid website | Pharmacy notifications | http://www.tn.gov/tenncare/pro-pharmacynotices.html |
|  | State Medicaid website | Events timeline | http://www.tn.gov/tenncare/news-timeline.html |
|  | State Medicaid website | Member pharmacy website | http://www.tn.gov/tenncare/mem-pharmacy.html |
|  | State secretary of state website | Medicaid rules and archive | http://www.tn.gov/sos/rules/tenncare.htm |
| Texas | State Medicaid website | Provider manuals and archive | <http://www.tmhp.com/Pages/Medicaid/Medicaid_Publications_Provider_manual.aspx> |
|  | State Medicaid website | Medicaid bulletins | <http://www.tmhp.com/Pages/Medicaid/medicaid_pubs_bulletin.aspx> |
| Utah | State Medicaid website | Provider manuals | http://health.utah.gov/medicaid/manuals/directory.php?p=Medicaid%20Provider%20Manuals/ |
|  | State Medicaid website | Information bulletins | http://health.utah.gov/medicaid/manuals/directory.php?p=Medicaid%20Information%20Bulletins/ |
| Vermont | State Medicaid website | Provider manuals | http://www.vtmedicaid.com/Downloads/manuals.html |
|  | State Medicaid website | Provider bulletins | http://www.vtmedicaid.com/Downloads/bulletins.html |
|  | State Medicaid website | Banner pages | http://www.vtmedicaid.com/Information/bannerarchives.html |
|  | Department of health access | Pharmacy benefit manual | http://dvha.vermont.gov/for-providers |
| Virginia | State Medicaid website | Provider manuals | https://www.virginiamedicaid.dmas.virginia.gov/wps/portal/ProviderManual |
|  | Regulatory town hall website | Town hall meeting minutes | http://townhall.virginia.gov/l/GetFile.cfm?File=E:\townhall\docroot\64\1618\3172\AgencyStatement_DMAS_3172_v2.pdf |
| Washington | State Medicaid website | Prescription drug program provider guide and archive | http://hrsa.dshs.wa.gov/download/Billing_Instructions_Webpages/Prescription_Drug_Program.html |
|  | State Medicaid website | Prescription drug related memoranda | http://hrsa.dshs.wa.gov/download/Billing_Instructions_Webpages/Prescription_Drug_Program.html |
|  | State Medicaid website | Medicaid PDL facts | http://hrsa.dshs.wa.gov/news/fact/FS007010PDLandPreauthfax0407update.pdf |
| West Virginia | Department of health and human resources website | Provider manuals | http://www.dhhr.wv.gov/bms/Pages/ProviderManuals.aspx |
|  | Direct contact with state Medicaid office |  |  |

**Online table 1 (continued). Sources of policy information organized by state**

| **State** | **Source** | **Section** | **Web Address** |
| --- | --- | --- | --- |
| Wisconsin | State Medicaid website | Provider updates and handbooks | <https://www.forwardhealth.wi.gov/WIPortal/Tab/42/icscontent/Provider/Updates/Index.htm.spage> |
|  | State Medicaid website | Pharmacy information | https://www.forwardhealth.wi.gov/WIPortal/Tab/42/icscontent/Provider/medicaid/pharmacy/resources.htm.spage |
| Wyoming | PBM website | Pharmacy manual | http://www.wyequalitycare.org/home/provider-manual?noCache=23:1329766253 |
|  | PBM website | Pharmacy notices and archive | http://www.wyequalitycare.org/pharmacy-info?noCache=318:1329766273 |
|  | State secretary of state website | State rules | http://soswy.state.wy.us/Rules/default.aspx |
|  | State Medicaid website | Provider bulletin | http://wyequalitycare.acs-inc.com/bulletins/pharm022599.pdf |

**Online table 2. Missing state policy information**

| **State** | **Missing information** |
| --- | --- |
| Arizona | All policies |
| Georgia | Exact date of 2005 cap removal |
| Nevada | Policies prior to 8/27/2004 |
| Tennessee | Policies prior to 7/1/2003 |
| Utah | Exact date of cap removal for traditional Medicaid recipients |

**Online table 3. Proportion of prescription usage for medications before and after cap implementation**

| **Overall cap implementation** | | | | | | | | | | | | | |
| --- | --- | --- | --- | --- | --- | --- | --- | --- | --- | --- | --- | --- | --- |
| *Relative Quarter* | *-6* | *-5* | *-4* | *-3* | *-2* | *-1* | *0* | *1* | *2* | *3* | *4* | *5* | *6* |
| Essential prescriptions | | | | | | | | | | | | | |
| Overall cap | 0.358 | 0.349 | 0.328 | 0.333 | 0.341 | 0.335 | 0.331 | 0.339 | 0.344 | 0.334 | 0.332 | 0.342 | 0.348 |
| Control | 0.414 | 0.409 | 0.407 | 0.412 | 0.418 | 0.413 | 0.415 | 0.419 | 0.426 | 0.421 | 0.423 | 0.428 | 0.434 |
| Essential expenditures | |  |  |  |  |  |  |  |  |  |  |  |  |
| Overall cap | 0.418 | 0.405 | 0.392 | 0.398 | 0.413 | 0.410 | 0.415 | 0.418 | 0.427 | 0.416 | 0.419 | 0.436 | 0.441 |
| Control | 0.442 | 0.443 | 0.437 | 0.444 | 0.453 | 0.452 | 0.456 | 0.461 | 0.470 | 0.471 | 0.476 | 0.482 | 0.491 |
| Preventive essential prescriptions | | | | | | | | | | | | | |
| Overall cap | 0.214 | 0.203 | 0.184 | 0.191 | 0.199 | 0.19 | 0.183 | 0.195 | 0.201 | 0.189 | 0.187 | 0.194 | 0.200 |
| Control | 0.244 | 0.240 | 0.238 | 0.243 | 0.249 | 0.245 | 0.247 | 0.252 | 0.258 | 0.255 | 0.257 | 0.262 | 0.269 |
| Preventive essential expenditures | | | | | | | | | | | | | |
| Overall cap | 0.186 | 0.172 | 0.156 | 0.158 | 0.164 | 0.155 | 0.151 | 0.158 | 0.164 | 0.153 | 0.152 | 0.155 | 0.159 |
| Control | 0.203 | 0.202 | 0.200 | 0.203 | 0.205 | 0.204 | 0.205 | 0.207 | 0.21 | 0.21 | 0.211 | 0.214 | 0.218 |
| Symptomatic essential prescriptions | | | | | | | | | | | | | |
| Overall cap | 0.145 | 0.146 | 0.144 | 0.142 | 0.143 | 0.145 | 0.147 | 0.143 | 0.143 | 0.145 | 0.146 | 0.149 | 0.147 |
| Control | 0.170 | 0.169 | 0.169 | 0.169 | 0.169 | 0.168 | 0.167 | 0.167 | 0.167 | 0.166 | 0.166 | 0.165 | 0.165 |
| Symptomatic essential expenditures | | | |  |  |  |  |  |  |  |  |  |  |
| Overall cap | 0.232 | 0.234 | 0.236 | 0.240 | 0.250 | 0.255 | 0.265 | 0.260 | 0.263 | 0.264 | 0.267 | 0.282 | 0.282 |
| Control | 0.239 | 0.242 | 0.236 | 0.241 | 0.247 | 0.248 | 0.251 | 0.254 | 0.260 | 0.261 | 0.264 | 0.268 | 0.273 |
| **Brand cap implementation** | | | | | | | | | | | | | |
| *Relative Quarter* | *-6* | *-5* | *-4* | *-3* | *-2* | *-1* | *0* | *1* | *2* | *3* | *4* | *5* | *6* |
| All brand prescriptions | | | | | | | | | | | | | |
| Brand cap | 0.525 | 0.524 | 0.519 | 0.514 | 0.508 | 0.492 | 0.476 | 0.463 | 0.456 | 0.445 | 0.433 | 0.428 | 0.424 |
| Control | 0.525 | 0.518 | 0.522 | 0.513 | 0.509 | 0.501 | 0.495 | 0.487 | 0.477 | 0.470 | 0.460 | 0.455 | 0.445 |
| All brand expenditures | | |  |  |  |  |  |  |  |  |  |  |  |
| Brand cap | 0.838 | 0.842 | 0.840 | 0.840 | 0.840 | 0.836 | 0.827 | 0.825 | 0.826 | 0.822 | 0.821 | 0.821 | 0.822 |
| Control | 0.845 | 0.848 | 0.850 | 0.845 | 0.846 | 0.842 | 0.843 | 0.843 | 0.840 | 0.841 | 0.835 | 0.835 | 0.836 |

**Online table 3 (continued). Proportion of prescription usage for medications before and after cap implementation**

| **Brand cap implementation** | | | | | | | | | | | | | |
| --- | --- | --- | --- | --- | --- | --- | --- | --- | --- | --- | --- | --- | --- |
| *Relative Quarter* | *-6* | *-5* | *-4* | *-3* | *-2* | *-1* | *0* | *1* | *2* | *3* | *4* | *5* | *6* |
| Selected classes: brand prescriptions^a^ | | | |  |  |  |  |  |  |  |  |  |  |
| Brand cap | 0.111 | 0.116 | 0.121 | 0.119 | 0.115 | 0.108 | 0.104 | 0.100 | 0.093 | 0.090 | 0.089 | 0.087 | 0.080 |
| Control | 0.120 | 0.119 | 0.122 | 0.118 | 0.117 | 0.113 | 0.113 | 0.108 | 0.103 | 0.097 | 0.095 | 0.092 | 0.086 |
| Selected classes: brand expenditures^a^ | | | |  |  |  |  |  |  |  |  |  |  |
| Brand cap | 0.173 | 0.180 | 0.183 | 0.183 | 0.177 | 0.165 | 0.157 | 0.152 | 0.144 | 0.141 | 0.140 | 0.135 | 0.126 |
| Control | 0.167 | 0.164 | 0.169 | 0.163 | 0.162 | 0.156 | 0.155 | 0.148 | 0.143 | 0.135 | 0.131 | 0.127 | 0.118 |
| Selected classes: generic prescriptions^a^ | | | | | | | | | | | | | |
| Brand cap | 0.056 | 0.057 | 0.054 | 0.057 | 0.058 | 0.064 | 0.069 | 0.070 | 0.071 | 0.072 | 0.074 | 0.075 | 0.075 |
| Control | 0.054 | 0.055 | 0.053 | 0.056 | 0.058 | 0.061 | 0.063 | 0.062 | 0.064 | 0.066 | 0.068 | 0.070 | 0.071 |
| Selected classes: generic expenditures^a^ | | | |  |  |  |  |  |  |  |  |  |  |
| Brand cap | 0.026 | 0.026 | 0.025 | 0.026 | 0.026 | 0.028 | 0.031 | 0.03 | 0.028 | 0.028 | 0.028 | 0.029 | 0.026 |
| Control | 0.027 | 0.026 | 0.024 | 0.026 | 0.026 | 0.026 | 0.026 | 0.024 | 0.025 | 0.025 | 0.025 | 0.025 | 0.024 |

^a^ Selected classes = ACE inhibitors, ARBs, CCBs, statins, NSAIDs, PPIs, SSRIs, and SNRIs

**Online table 4, A-L. Model parameter estimates**

**Online table 4A. Overall cap implementation: proportion of essential prescriptions**

| **Parameter** | **Estimate** | **Standard Error** | **95% Confidence interval** | | **Z-score** | **Pr > \|Z\|** |
| --- | --- | --- | --- | --- | --- | --- |
| Intercept | 0.4082 | 0.0032 | 0.4020 | 0.4145 | 128.1100 | <0.0001 |
| Time | 0.0009 | 0.0009 | -0.0009 | 0.0027 | 0.9700 | 0.3305 |
| Cap_state | -0.0766 | 0.0154 | -0.1068 | -0.0465 | -4.9800 | <0.0001 |
| Post_policy_ind | 0.0016 | 0.0036 | -0.0054 | 0.0086 | 0.4500 | 0.6548 |
| Post_policy_time | 0.0014 | 0.0010 | -0.0006 | 0.0034 | 1.3500 | 0.1781 |
| Sin(time) | 0.0010 | 0.0004 | 0.0002 | 0.0017 | 2.5900 | 0.0097 |
| Cos(time) | -0.0039 | 0.0003 | -0.0045 | -0.0033 | -12.7100 | <0.0001 |
| Sin(time)*cap_state | 0.0008 | 0.0015 | -0.0021 | 0.0038 | 0.5700 | 0.5689 |
| Cos(time)*cap_state | -0.0027 | 0.0013 | -0.0053 | -0.0001 | -2.0200 | 0.0430 |
| **Post_policy_ind*cap_state** | -0.0028 | 0.0024 | -0.0075 | 0.0019 | -1.1800 | 0.2372 |
| **Post_policy_time*cap_state** | -0.0013 | 0.0004 | -0.0021 | -0.0005 | -3.3700 | 0.0008 |

**Online table 4B. Overall cap implementation: proportion of essential expenditures**

| **Parameter** | **Estimate** | **Standard Error** | **95% Confidence interval** | | **Z-score** | **Pr > \|Z\|** |
| --- | --- | --- | --- | --- | --- | --- |
| Intercept | 0.4315 | 0.0063 | 0.4191 | 0.4439 | 68.1400 | <0.0001 |
| Time | 0.0031 | 0.0013 | 0.0005 | 0.0058 | 2.3400 | 0.0191 |
| Cap_state | -0.0398 | 0.0216 | -0.0821 | 0.0025 | -1.8400 | 0.0653 |
| Post_policy_ind | 0.0031 | 0.0062 | -0.0092 | 0.0153 | 0.4900 | 0.6236 |
| Post_policy_time | 0.0022 | 0.0013 | -0.0004 | 0.0048 | 1.6300 | 0.1041 |
| Sin(time) | -0.0004 | 0.0009 | -0.0022 | 0.0013 | -0.5000 | 0.6185 |
| Cos(time) | -0.0031 | 0.0005 | -0.0041 | -0.0021 | -6.0500 | <0.0001 |
| Sin(time)*cap_state | 0.0037 | 0.0023 | -0.0009 | 0.0083 | 1.5900 | 0.1126 |
| Cos(time)*cap_state | -0.0049 | 0.0030 | -0.0107 | 0.0009 | -1.6700 | 0.0953 |
| **Post_policy_ind*cap_state** | -0.0028 | 0.0063 | -0.0152 | 0.0097 | -0.4400 | 0.6619 |
| **Post_policy_time*cap_state** | -0.0017 | 0.0025 | -0.0066 | 0.0032 | -0.6900 | 0.4921 |

**Online table 4C. Overall cap implementation: proportion of preventive essential prescriptions**

| **Parameter** | **Estimate** | **Standard Error** | **95% Confidence interval** | | **Z-score** | **Pr > \|Z\|** |
| --- | --- | --- | --- | --- | --- | --- |
| Intercept | 0.2381 | 0.0036 | 0.2310 | 0.2452 | 65.8000 | <0.0001 |
| Time | 0.0010 | 0.0009 | -0.0006 | 0.0027 | 1.2100 | 0.2257 |
| Cap_state | -0.0499 | 0.0130 | -0.0754 | -0.0244 | -3.8400 | 0.0001 |
| Post_policy_ind | 0.0032 | 0.0036 | -0.0040 | 0.0103 | 0.8700 | 0.3829 |
| Post_policy_time | 0.0018 | 0.0010 | -0.0001 | 0.0037 | 1.8300 | 0.0669 |
| Sin(time) | 0.0009 | 0.0003 | 0.0003 | 0.0015 | 2.9500 | 0.0032 |
| Cos(time) | -0.0035 | 0.0005 | -0.0045 | -0.0025 | -6.7600 | <0.0001 |
| Sin(time)*cap_state | 0.0009 | 0.0017 | -0.0023 | 0.0042 | 0.5700 | 0.5718 |
| Cos(time)*cap_state | -0.0037 | 0.0016 | -0.0069 | -0.0004 | -2.2300 | 0.0255 |
| **Post_policy_ind*cap_state** | -0.0047 | 0.0029 | -0.0105 | 0.0010 | -1.6200 | 0.1050 |
| **Post_policy_time*cap_state** | -0.0028 | 0.0009 | -0.0046 | -0.0011 | -3.2300 | 0.0012 |

**Online table 4D. Overall cap implementation: proportion of preventive essential expenditures**

| **Parameter** | **Estimate** | **Standard Error** | **95% Confidence interval** | | **Z-score** | **Pr > \|Z\|** |
| --- | --- | --- | --- | --- | --- | --- |
| Intercept | 0.2058 | 0.0031 | 0.1996 | 0.2119 | 65.5900 | <0.0001 |
| Time | -0.0012 | 0.0010 | -0.0032 | 0.0008 | -1.1700 | 0.2426 |
| Cap_state | -0.0385 | 0.0164 | -0.0707 | -0.0063 | -2.3400 | 0.0191 |
| Post_policy_ind | 0.0070 | 0.0042 | -0.0012 | 0.0153 | 1.6700 | 0.0939 |
| Post_policy_time | 0.0033 | 0.0010 | 0.0014 | 0.0052 | 3.3400 | 0.0008 |
| Sin(time) | 0.0001 | 0.0003 | -0.0004 | 0.0006 | 0.5500 | 0.5836 |
| Cos(time) | -0.0015 | 0.0004 | -0.0023 | -0.0007 | -3.6700 | 0.0002 |
| Sin(time)*cap_state | 0.0010 | 0.0018 | -0.0025 | 0.0044 | 0.5600 | 0.5727 |
| Cos(time)*cap_state | -0.0044 | 0.0023 | -0.0088 | 0.0000 | -1.9600 | 0.0504 |
| **Post_policy_ind*cap_state** | -0.0044 | 0.0046 | -0.0135 | 0.0046 | -0.9600 | 0.3372 |
| **Post_policy_time*cap_state** | -0.0030 | 0.0007 | -0.0043 | -0.0017 | -4.4100 | <0.0001 |

**Online table 4E. Overall cap implementation: proportion of symptomatic essential prescriptions**

| **Parameter** | **Estimate** | **Standard Error** | **95% Confidence interval** | | **Z-score** | **Pr > \|Z\|** |
| --- | --- | --- | --- | --- | --- | --- |
| Intercept | 0.1705 | 0.0012 | 0.1681 | 0.1730 | 137.7800 | <0.0001 |
| Time | -0.0003 | 0.0002 | -0.0007 | 0.0002 | -1.1800 | 0.2373 |
| Cap_state | -0.0265 | 0.0084 | -0.0431 | -0.0100 | -3.1500 | 0.0016 |
| Post_policy_ind | -0.0012 | 0.0008 | -0.0027 | 0.0004 | -1.4300 | 0.1533 |
| Post_policy_time | -0.0002 | 0.0002 | -0.0007 | 0.0002 | -0.9600 | 0.3348 |
| Sin(time) | 0.0001 | 0.0001 | -0.0001 | 0.0003 | 0.8500 | 0.3951 |
| Cos(time) | -0.0005 | 0.0002 | -0.0009 | 0.0000 | -2.0500 | 0.0401 |
| Sin(time)*cap_state | -0.0001 | 0.0008 | -0.0016 | 0.0014 | -0.1300 | 0.9003 |
| Cos(time)*cap_state | 0.0009 | 0.0005 | -0.0001 | 0.0019 | 1.7700 | 0.0767 |
| **Post_policy_ind*cap_state** | 0.0019 | 0.0006 | 0.0007 | 0.0031 | 3.0500 | 0.0023 |
| **Post_policy_time*cap_state** | 0.0014 | 0.0010 | -0.0005 | 0.0034 | 1.4200 | 0.1542 |

**Online table 4F. Overall cap implementation: proportion of symptomatic essential expenditures**

| **Parameter** | **Estimate** | **Standard Error** | **95% Confidence interval** | | **Z-score** | **Pr > \|Z\|** |
| --- | --- | --- | --- | --- | --- | --- |
| Intercept | 0.2259 | 0.0067 | 0.2128 | 0.2389 | 33.9200 | <0.0001 |
| Time | 0.0043 | 0.0013 | 0.0017 | 0.0068 | 3.2800 | 0.0010 |
| Cap_state | -0.0012 | 0.0335 | -0.0669 | 0.0645 | -0.0400 | 0.9714 |
| Post_policy_ind | -0.0037 | 0.0045 | -0.0126 | 0.0051 | -0.8200 | 0.4095 |
| Post_policy_time | -0.0010 | 0.0014 | -0.0038 | 0.0018 | -0.6900 | 0.4912 |
| Sin(time) | -0.0006 | 0.0006 | -0.0018 | 0.0007 | -0.9200 | 0.3575 |
| Cos(time) | -0.0016 | 0.0002 | -0.0020 | -0.0011 | -6.8000 | <0.0001 |
| Sin(time)*cap_state | 0.0027 | 0.0020 | -0.0013 | 0.0067 | 1.3300 | 0.1820 |
| Cos(time)*cap_state | -0.0006 | 0.0008 | -0.0021 | 0.0009 | -0.7200 | 0.4702 |
| **Post_policy_ind*cap_state** | 0.0015 | 0.0023 | -0.0031 | 0.0061 | 0.6500 | 0.5179 |
| **Post_policy_time*cap_state** | 0.0011 | 0.0031 | -0.0049 | 0.0071 | 0.3600 | 0.7186 |

**Online table 4G. Brand cap implementation: proportion of brand prescriptions**

| **Parameter** | **Estimate** | **Standard Error** | **95% Confidence interval** | | **Z-score** | **Pr > \|Z\|** |
| --- | --- | --- | --- | --- | --- | --- |
| Intercept | 0.5415 | 0.0190 | 0.5043 | 0.5787 | 28.5400 | <0.0001 |
| Time | -0.0059 | 0.0009 | -0.0077 | -0.0042 | -6.5400 | <0.0001 |
| Cap_state | -0.0067 | 0.0281 | -0.0619 | 0.0485 | -0.2400 | 0.8115 |
| Post_policy_ind | -0.0028 | 0.0030 | -0.0087 | 0.0031 | -0.9200 | 0.3571 |
| Post_policy_time | -0.0020 | 0.0009 | -0.0039 | -0.0001 | -2.1100 | 0.0348 |
| Part_D | -0.0075 | 0.0019 | -0.0112 | -0.0039 | -4.0400 | <0.0001 |
| Sin(time) | 0.0002 | 0.0005 | -0.0007 | 0.0012 | 0.4400 | 0.6591 |
| Cos(time) | 0.0023 | 0.0004 | 0.0016 | 0.0031 | 6.1900 | <0.0001 |
| Part_D*cap_state | 0.0093 | 0.0098 | -0.0098 | 0.0284 | 0.9600 | 0.3389 |
| Sin(time)*cap_state | 0.0000 | 0.0012 | -0.0024 | 0.0024 | -0.0200 | 0.9849 |
| Cos(time)*cap_state | 0.0025 | 0.0020 | -0.0015 | 0.0064 | 1.2300 | 0.2184 |
| **Post_policy_ind*cap_state** | -0.0229 | 0.0095 | -0.0416 | -0.0042 | -2.4000 | 0.0162 |
| **Post_policy_time*cap_state** | -0.0002 | 0.0010 | -0.0023 | 0.0018 | -0.2100 | 0.8340 |

**Online table 4H. Brand cap implementation: proportion of brand expenditures**

| **Parameter** | **Estimate** | **Standard Error** | **95% Confidence interval** | | **Z-score** | **Pr > \|Z\|** |
| --- | --- | --- | --- | --- | --- | --- |
| Intercept | 0.8473 | 0.0050 | 0.8375 | 0.8570 | 169.6600 | <0.0001 |
| Time | -0.0003 | 0.0006 | -0.0015 | 0.0010 | -0.4500 | 0.6540 |
| Cap_state | -0.0086 | 0.0116 | -0.0312 | 0.0141 | -0.7400 | 0.4590 |
| Post_policy_ind | -0.0027 | 0.0032 | -0.0089 | 0.0036 | -0.8400 | 0.3983 |
| Post_policy_time | -0.0018 | 0.0012 | -0.0041 | 0.0005 | -1.5600 | 0.1185 |
| Part_D | 0.0079 | 0.0018 | 0.0044 | 0.0114 | 4.4200 | <0.0001 |
| Sin(time) | 0.0023 | 0.0007 | 0.0008 | 0.0037 | 3.1000 | 0.0019 |
| Cos(time) | 0.0008 | 0.0006 | -0.0003 | 0.0019 | 1.4400 | 0.1510 |
| Part_D*cap_state | 0.0013 | 0.0087 | -0.0158 | 0.0184 | 0.1500 | 0.8796 |
| Sin(time)*cap_state | -0.0014 | 0.0009 | -0.0032 | 0.0004 | -1.5600 | 0.1177 |
| Cos(time)*cap_state | 0.0009 | 0.0009 | -0.0009 | 0.0026 | 0.9900 | 0.3222 |
| **Post_policy_ind*cap_state** | -0.0126 | 0.0056 | -0.0236 | -0.0016 | -2.2400 | 0.0249 |
| **Post_policy_time*cap_state** | 0.0008 | 0.0014 | -0.0019 | 0.0035 | 0.5900 | 0.5551 |

**Online table 4I. Brand cap implementation: classes with generic replacements, proportion of brand prescriptions**

| **Parameter** | **Estimate** | **Standard Error** | **95% Confidence interval** | | **Z-score** | **Pr > \|Z\|** |
| --- | --- | --- | --- | --- | --- | --- |
| Intercept | 0.1281 | 0.0053 | 0.1177 | 0.1385 | 0.2413 | <0.0001 |
| Time | -0.0013 | 0.0006 | -0.0025 | -0.0001 | -0.0217 | 0.0298 |
| Cap_state | -0.0055 | 0.0110 | -0.0270 | 0.0161 | -0.0050 | 0.6194 |
| Post_policy_ind | -0.0013 | 0.0016 | -0.0045 | 0.0018 | -0.0083 | 0.4069 |
| Post_policy_time | -0.0019 | 0.0011 | -0.0040 | 0.0003 | -0.0173 | 0.0839 |
| Part_D | -0.0202 | 0.0014 | -0.0230 | -0.0175 | -0.1431 | <0.0001 |
| Sin(time) | 0.0002 | 0.0003 | -0.0004 | 0.0008 | 0.0062 | 0.5329 |
| Cos(time) | -0.0010 | 0.0003 | -0.0017 | -0.0003 | -0.0291 | 0.0036 |
| Part_D*cap_state | 0.0063 | 0.0024 | 0.0017 | 0.0110 | 0.0267 | 0.0076 |
| Sin(time)*cap_state | 0.0004 | 0.0009 | -0.0013 | 0.0021 | 0.0045 | 0.6544 |
| Cos(time)*cap_state | -0.0002 | 0.0010 | -0.0021 | 0.0018 | -0.0016 | 0.8713 |
| **Post_policy_ind*cap_state** | -0.0074 | 0.0025 | -0.0123 | -0.0025 | -0.0295 | 0.0031 |
| **Post_policy_time*cap_state** | 0.0002 | 0.0012 | -0.0022 | 0.0026 | 0.0015 | 0.8805 |

**Online table 4J. Brand cap implementation classes with generic replacements, proportion of brand expenditures**

| **Parameter** | **Estimate** | **Standard Error** | **95% Confidence interval** | | **Z-score** | **Pr > \|Z\|** |
| --- | --- | --- | --- | --- | --- | --- |
| Intercept | 0.1807 | 0.0085 | 0.1639 | 0.1974 | 0.2114 | <0.0001 |
| Time | -0.0023 | 0.0008 | -0.0038 | -0.0009 | -0.0310 | 0.0020 |
| Cap_state | 0.0110 | 0.0176 | -0.0236 | 0.0455 | 0.0062 | 0.5338 |
| Post_policy_ind | -0.0016 | 0.0028 | -0.0071 | 0.0040 | -0.0055 | 0.5840 |
| Post_policy_time | -0.0016 | 0.0016 | -0.0048 | 0.0016 | -0.0099 | 0.3208 |
| Part_D | -0.0321 | 0.0022 | -0.0363 | -0.0278 | -0.1473 | <0.0001 |
| Sin(time) | -0.0003 | 0.0003 | -0.0010 | 0.0004 | -0.0088 | 0.3771 |
| Cos(time) | -0.0009 | 0.0004 | -0.0016 | -0.0002 | -0.0247 | 0.0137 |
| Part_D*cap_state | 0.0068 | 0.0071 | -0.0071 | 0.0207 | 0.0096 | 0.3381 |
| Sin(time)*cap_state | -0.0003 | 0.0010 | -0.0023 | 0.0017 | -0.0033 | 0.7450 |
| Cos(time)*cap_state | -0.0010 | 0.0014 | -0.0037 | 0.0018 | -0.0067 | 0.5022 |
| **Post_policy_ind*cap_state** | -0.0127 | 0.0038 | -0.0201 | -0.0053 | -0.0337 | 0.0007 |
| **Post_policy_time*cap_state** | 0.0005 | 0.0018 | -0.0029 | 0.0040 | 0.0030 | 0.7670 |

**Online table 4K. Brand cap implementation: classes with generic replacements, proportion of generic prescriptions**

| **Parameter** | **Estimate** | **Standard Error** | **95% Confidence interval** | | **Z-score** | **Pr > \|Z\|** |
| --- | --- | --- | --- | --- | --- | --- |
| Intercept | 0.0491 | 0.0070 | 0.0354 | 0.0629 | 0.0700 | <0.0001 |
| Time | 0.0021 | 0.0003 | 0.0016 | 0.0026 | 0.0822 | <0.0001 |
| Cap_state | 0.0012 | 0.0116 | -0.0216 | 0.0240 | 0.0010 | 0.9188 |
| Post_policy_ind | -0.0005 | 0.0011 | -0.0026 | 0.0016 | -0.0045 | 0.6561 |
| Post_policy_time | 0.0005 | 0.0006 | -0.0006 | 0.0016 | 0.0092 | 0.3590 |
| Part_D | -0.0103 | 0.0014 | -0.0131 | -0.0075 | -0.0727 | <0.0001 |
| Sin(time) | -0.0001 | 0.0002 | -0.0005 | 0.0004 | -0.0038 | 0.7018 |
| Cos(time) | -0.0002 | 0.0001 | -0.0004 | 0.0000 | -0.0202 | 0.0438 |
| Part_D*cap_state | -0.0009 | 0.0048 | -0.0103 | 0.0086 | -0.0018 | 0.8603 |
| Sin(time)*cap_state | -0.0004 | 0.0005 | -0.0013 | 0.0005 | -0.0092 | 0.3579 |
| Cos(time)*cap_state | -0.0006 | 0.0004 | -0.0014 | 0.0002 | -0.0144 | 0.1493 |
| **Post_policy_ind*cap_state** | 0.0079 | 0.0030 | 0.0020 | 0.0138 | 0.0263 | 0.0085 |
| **Post_policy_time*cap_state** | -0.0008 | 0.0006 | -0.0019 | 0.0003 | -0.0148 | 0.1400 |

**Online table 4L. Brand cap implementation classes with generic replacements, proportion of generic expenditures**

| **Parameter** | **Estimate** | **Standard Error** | **95% Confidence interval** | | **Z-score** | **Pr > \|Z\|** |
| --- | --- | --- | --- | --- | --- | --- |
| Intercept | 0.0246 | 0.0026 | 0.0195 | 0.0298 | 0.0933 | <0.0001 |
| Time | 0.0004 | 0.0004 | -0.0004 | 0.0012 | 0.0093 | 0.3505 |
| Cap_state | 0.0006 | 0.0040 | -0.0072 | 0.0084 | 0.0015 | 0.8821 |
| Post_policy_ind | -0.0017 | 0.0022 | -0.0060 | 0.0026 | -0.0076 | 0.4472 |
| Post_policy_time | -0.0001 | 0.0007 | -0.0014 | 0.0012 | -0.0009 | 0.9263 |
| Part_D | -0.0051 | 0.0013 | -0.0077 | -0.0025 | -0.0380 | 0.0001 |
| Sin(time) | -0.0007 | 0.0002 | -0.0010 | -0.0003 | -0.0334 | 0.0008 |
| Cos(time) | 0.0000 | 0.0002 | -0.0004 | 0.0004 | 0.0005 | 0.9586 |
| Part_D*cap_state | -0.0054 | 0.0052 | -0.0157 | 0.0049 | -0.0102 | 0.3057 |
| Sin(time)*cap_state | 0.0002 | 0.0003 | -0.0004 | 0.0008 | 0.0060 | 0.5494 |
| Cos(time)*cap_state | -0.0001 | 0.0005 | -0.0010 | 0.0007 | -0.0031 | 0.7535 |
| **Post_policy_ind*cap_state** | 0.0060 | 0.0037 | -0.0012 | 0.0131 | 0.0163 | 0.1022 |
| **Post_policy_time*cap_state** | -0.0003 | 0.0005 | -0.0013 | 0.0008 | -0.0048 | 0.6298 |

**Online Figure 1. Proportion of prescriptions and spending accounted for by brand drugs before and after implementation of brand cap policies**

1. Prescriptions

**
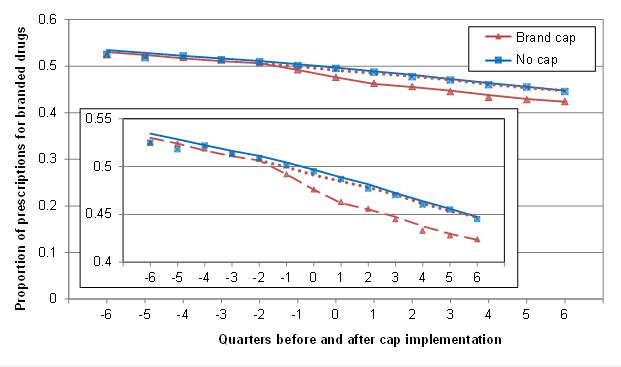
**

Start of cap policy

1. Spending


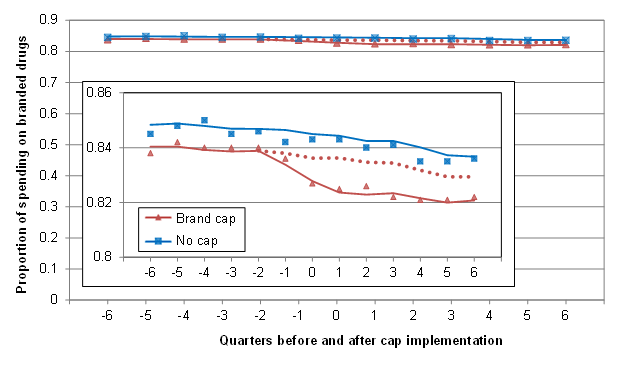
Triangles and squares represent measured proportion of utilization. Solid lines represent predicted utilization based on models. The dotted line represents predicted utilization if brand cap policies had not been implemented (the counterfactual). Time is measured in calendar quarters relative to policy implementation. The weighted average of medication use in states without prescription caps throughout the study period was used as a control. The time frame for the control data was standardized relative to the quarter in which the cap policy was initiated in the intervention state.

Start of cap policy
